# Physiological and Transcriptomic Dissection of Inflorescence Degeneration in *Areca catechu* L.: Aberrant Carbohydrate Redistribution and Disrupted Hormonal Homeostasis

**DOI:** 10.3390/plants15131962

**Published:** 2026-06-25

**Authors:** Weike Yao, Han Li, Meng Tian, Shanyue Rong, Chao Ma, Ruping Li, Hanying Zhang, Fusun Yang, Changzhen Li

**Affiliations:** School of Tropical Agriculture and Forestry, Hainan University, Haikou 570228, China; yaoweike2024@163.com (W.Y.); lihann123@163.com (H.L.); 23220951310004@hainanu.edu.cn (M.T.); 18389476613@163.com (S.R.); 18156381357@163.com (C.M.); m18336715663@163.com (R.L.); 23220951310166@hainanu.edu.cn (H.Z.)

**Keywords:** *Areca catechu*, inflorescence degeneration, carbohydrate metabolism, phytohormone signaling, transcriptome, WGCNA

## Abstract

Inflorescence degeneration in *Areca catechu* L. is characterized by growth arrest, tissue shrinkage and browning, ultimately compromising functional inflorescence formation and yield stability. To investigate its developmental window and regulatory basis, inflorescences from different leaf positions at the full-bloom stage were analyzed using anatomical observation, morphological measurements, carbohydrate and hormone assays, and RNA-seq-based transcriptomic analysis with qRT-PCR validation. Inflorescence degeneration was mainly concentrated in axillary inflorescences at the third and fourth leaf positions (BY3 and BY4). Compared with adjacent normal inflorescences, degenerated inflorescences showed reduced sucrose, starch and trehalose contents, increased ABA, JA and MeJA levels, and decreased cZR levels. Transcriptomic analysis revealed clear separation between degenerated and normal inflorescences, and differentially expressed genes were enriched in starch and sucrose metabolism, plant hormone signal transduction and transcriptional regulation. Co-expression network analysis identified modules associated with the degeneration window and key physiological traits, highlighting six candidate hub genes: *AcAHP2*, *AcTIFY4B*, *AcTPS9-2*, *AcHXK2*, *AcWRKY3* and *AcMPK1*. These findings suggest that inflorescence degeneration is closely associated with carbon metabolic imbalance, hormone network remodeling and co-expression network reprogramming within a specific developmental window, providing a basis for future mechanistic studies and control strategies.

## 1. Introduction

Areca palm (*Areca catechu* L.), a member of the genus Areca in the family Arecaceae, is regarded as the foremost of China’s four major southern medicinal plants and is an important tropical economic crop in Hainan Province [1]. Its spadix-type inflorescences, enclosed by spathes, emerge synchronously with leaves, with one inflorescence enclosed in the axil of each leaf. As new leaves emerge, new inflorescences are formed, whereas the spathe surrounding an inflorescence opens after the associated older leaf abscises. This developmental feature provides a morphological basis for identifying inflorescence developmental progression according to leaf position [2]. However, some inflorescences undergo degeneration during this process, characterized by growth arrest, tissue shrinkage and browning, and failure to develop into functional inflorescences, thereby restricting year-round flowering and limiting yield improvement (Figure S1) [3]. From the perspective of plant reproductive development, these abnormalities, involving growth cessation, structural regression and developmental arrest during organ formation, are comparable to inflorescence degeneration, floral bud abortion, spikelet abortion and organ abscission reported in other plant species [4,5,6]. Based on the phenotypic characteristics observed in this study, this phenomenon is hereafter collectively referred to as “inflorescence degeneration.”

Abnormal development of plant reproductive organs does not usually occur at random but is often concentrated at specific developmental stages that are highly sensitive to nutrient supply, environmental factors and endogenous regulation [7]. For example, such abnormalities may occur during the 4–6 months before maturation [8], during the key morphogenetic stage of sex differentiation [9], or during the critical period of bud-scale separation at the late stage of floral bud development [10]. These findings indicate that abnormal development of inflorescences or spikes in different plant species commonly exhibits stage-specific characteristics rather than occurring randomly. Therefore, accurately defining the critical window in which abnormal development occurs is an important prerequisite for elucidating its underlying mechanisms.

Once the critical window has been identified, clarifying the intrinsic drivers of inflorescence degeneration becomes a central research focus. Previous studies have shown that the transition of inflorescences or floral buds from normal development to degeneration is not caused by a single factor, but results from the combined effects of developmental programs, resource allocation, and endogenous signaling [5,11,12]. On the one hand, non-structural carbohydrates such as sucrose and starch not only provide energy for floral organ formation but also act as signaling molecules that regulate developmental progression. Carbohydrate accumulation in branches without timely transport to floral buds [13], an imbalanced proportion of different carbohydrate forms [14], and disruptions in source-sink relationships and carbon-flow allocation can collectively shift organ development from sustained construction toward stagnation or degeneration [15]. In addition, sugar signaling reflects not only the level of carbon reserves, but can also regulate developmental trajectories through sucrose metabolism and the Tre6P/TPS signaling module [16], thereby promoting a transition from organ formation toward senescence- and inhibition-associated programs. These physiological changes are closely associated with aberrant expression of genes involved in carbohydrate metabolism. In rice, downregulation of the invertase gene *OsCWIN3* and the transporter genes *OsSUT5*, *OsMST1*, and *OsMST8* directly impairs sugar utilization or leads to abnormal sugar accumulation [14,17]. Similarly, low expression of *CWI2* in bisexual flowers of *Tapiscia sinensis* is accompanied by marked reductions in starch and soluble sugar contents [18]. These findings indicate that transcriptional reprogramming of key carbohydrate metabolism genes constitutes an important molecular basis for abnormal carbohydrate redistribution. On the other hand, dynamic hormonal balance, particularly the accumulation of abscisic acid (ABA) and jasmonic acid (JA), together with a deficiency of cytokinins such as cis-zeatin riboside (cZR), has been shown to induce floral organ developmental arrest or premature abscission [19,20]. In abortive floral buds, ABA content increases significantly and induces programmed cell death [12], whereas expression of the cytokinin biosynthetic gene IPT in maize can reverse pistil abortion [21]. Hormonal changes are likewise governed by finely tuned gene expression networks. ABA may function both in promoting reproductive abortion and in regulating floral movement. In lily, ABA accumulation caused by upregulation of *LoNCED* suppresses tapetum-related genes and ultimately leads to pollen abortion [22]. In rice, *OsNCED3* and *OsPYL1* positively regulate spikelet closure through ABA signaling [23]. Regarding JA, this hormone is essential for male floral development; impaired JA biosynthesis prevents programmed cell death in pistils and results in seed formation on tassels [24]. Disruption of the JA regulatory module MSD1-MSD2 also compromises normal spikelet development [25]. For cytokinins, significantly elevated cytokinin levels have been observed in abortive floral buds and were shown to inhibit normal pistil development [26]. Although this appears inconsistent with the conclusion that cytokinin deficiency can lead to reproductive abortion, these findings collectively indicate that disruption of cytokinin homeostasis—whether through excessive accumulation or insufficient levels—can interfere with floral organ developmental programs [21]. In summary, abnormal carbohydrate redistribution and disrupted hormonal homeostasis are not isolated events. Instead, they are coordinated through complex gene expression networks and jointly drive the progression of inflorescence degeneration.

In recent years, studies on reproductive development in areca palm have provided a foundation in floral organ differentiation, sex determination, and organ abscission. Previous studies have shown that hormone signaling pathways involving JA and IAA, together with MADS-box transcription factors, participate in the regulation of floral organ development and female and male flower differentiation in areca palm [27]. In addition, field diseases are important external factors affecting plant health and yield stability in areca palm. Related studies have isolated and identified multiple fungal pathogens from diseased areca palm tissues in Hainan Island, suggesting that pathogen infection may represent a potential external stress factor affecting the health status of areca palm reproductive organs [28]. However, the developmental basis and regulatory mechanisms underlying the commonly observed phenomenon of inflorescence degeneration in production remain insufficiently understood; in particular, whether these mechanisms are concentrated within a specific developmental window remains unclear. Therefore, preliminary field investigations and anatomical observations were conducted to identify an appropriate phenological stage for studying areca palm. Using areca palm inflorescences at different leaf positions as experimental materials, we defined the high-incidence stage of degeneration through anatomical observation and further integrated morphological measurements, endogenous hormone and carbohydrate content analyses, transcriptome sequencing-based identification of differentially expressed genes, WGCNA and qRT-PCR validation to address the following questions: Does areca palm inflorescence degeneration occur within a clearly defined high-incidence window? Do carbohydrates and hormones exhibit directional changes within this window? Do these changes converge at the transcriptomic level into key pathways, modules and hub genes? These findings will deepen our understanding of the mechanisms underlying areca palm inflorescence degeneration from the perspectives of developmental windows, physiological imbalance and network regulation, and provide a theoretical basis for anti-degeneration regulation and germplasm improvement.

## 2. Results

### 2.1. Identification of the Inflorescence Degeneration Window in Areca Palm Based on Morphological Characteristics

To determine the appropriate sampling period, preliminary anatomical observations of inflorescence primordia at different leaf positions were conducted during the flowering stage in March, the full-bloom stage in June, and the fruiting stage in September 2025. The results showed that no obvious degeneration had occurred in March, whereas by September, degeneration had extended to the L1 and L2 leaf positions, making both stages unsuitable for comparative analysis between degenerated inflorescences and adjacent normally developing inflorescences (Figure S2). In contrast, degeneration in June was mainly concentrated at the BY3 and BY4 leaf positions, while the adjacent leaf positions on both sides still maintained normal development. Therefore, samples collected during the full-bloom stage in June 2025 were selected for subsequent analyses.

**Figure 1:**
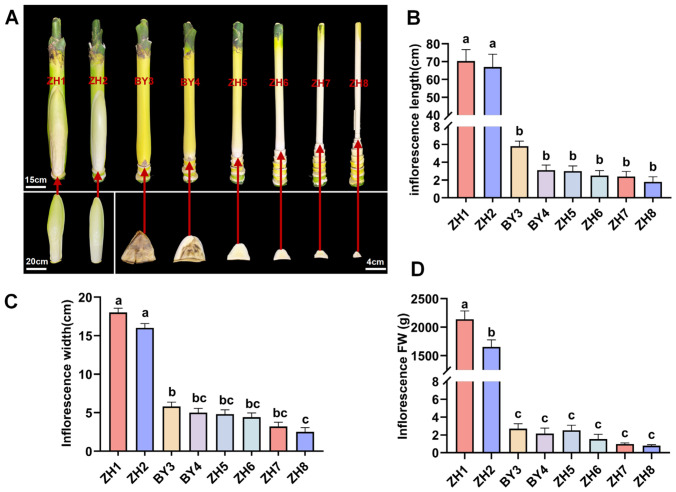
Morphological characteristics of areca palm inflorescences. (**A**) External morphology and corresponding anatomical status of inflorescences at different leaf positions. Based on the sampling time point, leaf positions were sequentially numbered from outer to inner as ZH1, ZH2, BY3, BY4, ZH5, ZH6, ZH7, and ZH8. Red arrows indicate the positions of inflorescences at the corresponding leaf positions. BY3 and BY4 inflorescences exhibited obvious degeneration phenotypes, including blackening, shrinkage, and developmental arrest. (**B**–**D**) Length (**B**), width (**C**), and fresh weight (**D**) of inflorescences at different leaf positions. Data are presented as the mean ± SD (*n* = 5). Different lowercase letters indicate significant differences among leaf positions (*p* < 0.05). Scale bars are indicated in the figure.

To clarify the occurrence position and morphological characteristics of degenerated inflorescences during this period, the length, width and fresh weight of inflorescences at different leaf positions were first measured. The results showed that the length, width and fresh weight of ZH1 and ZH2 inflorescences were maintained at relatively high levels, with no obvious abnormalities observed. In contrast, BY3 and BY4 inflorescences showed significant decreases in length, width and fresh weight, and the inflorescences were visibly reduced in size. Inflorescences located at the more inner leaf positions from ZH5 to ZH8 were generally smaller, but their tissue color remained normal and no obvious abnormalities were observed (Figure 1A–D). In addition, compared with normally developing inflorescences, BY3 and BY4 inflorescences exhibited typical degeneration phenotypes, including browning of the inflorescence buds, tissue shrinkage and developmental arrest (Figure 1A). Taken together, during the sampling period of this study, inflorescence degeneration in areca palm showed a clearly defined occurrence window, mainly concentrated in BY3 and BY4 inflorescences at the full-bloom stage. Accordingly, BY3 and BY4 were used as the degeneration window, while ZH2 and ZH5 were used as adjacent controls for subsequent analyses of hormones, carbohydrates and transcriptomic changes.

### 2.2. Changes in Carbohydrate Contents Within the Degeneration Window

**Figure 2:**
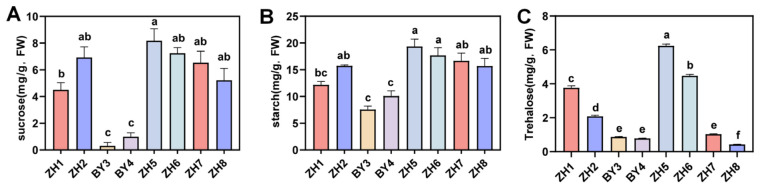
Changes in carbohydrate contents within the degeneration window. (**A**–**C**) Changes in sucrose (**A**), starch (**B**), and trehalose (**C**) contents in areca palm inflorescences at different developmental stages. Data are presented as the mean ± SD (*n* = 3). Different lowercase letters indicate significant differences among developmental stages (one-way ANOVA followed by Tukey’s test, *p* < 0.05).

To further characterize the carbohydrate changes during the degeneration window of areca palm inflorescences, and considering the roles of sucrose in assimilate transport, starch in carbon storage, and the trehalose signaling module in sugar status sensing, the contents of sucrose, starch, and trehalose were measured in inflorescences at different developmental stages. The results showed that sucrose and starch contents gradually increased during the earlier developmental stages from ZH8 to ZH5 but decreased during the later developmental stages from ZH2 to ZH1 (Figure 2A,B). Within the degeneration window, sucrose content in BY4 and BY3 was significantly lower than that in the adjacent normally developing inflorescences ZH5 and ZH2. Specifically, sucrose content in BY4 decreased by 87.90% and 85.73% compared with ZH5 and ZH2, respectively, while that in BY3 decreased by 96.28% and 95.61%, respectively (Figure 2A). The pattern of starch content was generally consistent with that of sucrose. Compared with ZH5 and ZH2, starch content in BY4 decreased by 47.85% and 35.79%, respectively, while that in BY3 decreased by 61.03% and 51.98%, respectively (Figure 2B). Trehalose content also decreased significantly within the degeneration window. Compared with ZH5 and ZH2, trehalose content in BY4 decreased by 87.50% and 62.50%, respectively, while that in BY3 decreased by 86.22% and 58.65%, respectively (Figure 2C).

### 2.3. Changes in Endogenous Hormone Profiles Within the Degeneration Window

**Figure 3:**
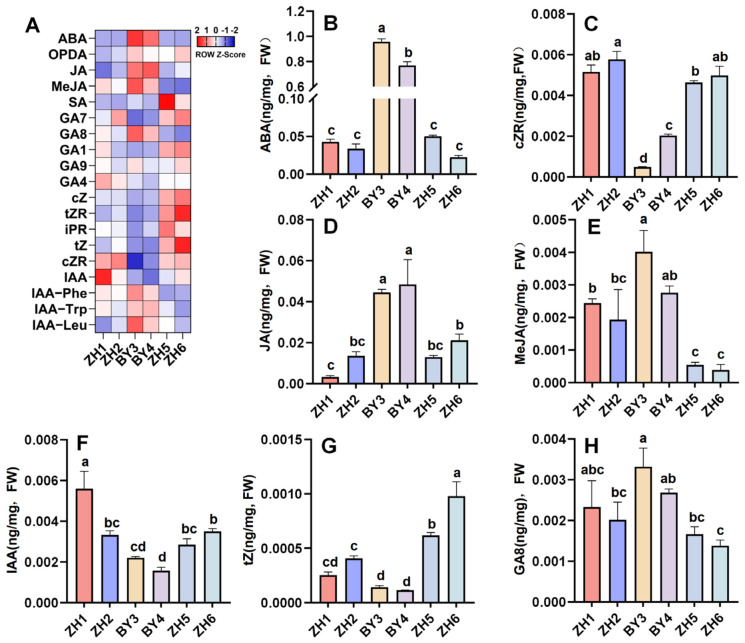
Changes in phytohormones and related metabolites within the degeneration window. (**A**) Heatmap showing the levels of 19 endogenous phytohormones and related metabolites in areca palm inflorescences at different developmental stages. Heatmap colors represent the relative accumulation levels of each hormone or metabolite at different stages, with row-standardized data shown as row Z-score values. (**B**–**H**) Comparisons of abscisic acid (ABA, (**B**)), cis-zeatin riboside (cZR, (**C**)), jasmonic acid (JA, (**D**)), methyl jasmonate (MeJA, (**E**)), indole-3-acetic acid (IAA, (**F**)), trans-zeatin (tZ, (**G**)), and gibberellin A8 (GA8, (**H**)) contents in areca palm inflorescences at different developmental stages. Data are presented as the mean ± SD (*n* = 3). Different lowercase letters indicate significant differences among developmental stages (one-way ANOVA followed by Tukey’s test, *p* < 0.05).

To characterize hormonal regulatory features associated with the window of areca palm inflorescence degeneration, 19 endogenous phytohormones and related metabolites detected in inflorescences at different developmental stages were quantitatively analyzed. The results showed that abscisic acid (ABA), 12-oxo-phytodienoic acid (OPDA), jasmonic acid (JA), methyl jasmonate (MeJA), gibberellin A8 (GA8), and several auxin-conjugated metabolites, including indole-3-acetyl-L-phenylalanine (IAA-Phe), indole-3-acetyl-L-tryptophan (IAA-Trp), and indole-3-acetyl-L-leucine (IAA-Leu), generally showed increasing trends within the degeneration window (Figure 3A). In contrast, gibberellins GA1, GA4, and GA7, indole-3-acetic acid (IAA), and several cytokinin components, including cis-zeatin (cZ), trans-zeatin (tZ), cis-zeatin riboside (cZR), trans-zeatin riboside (tZR), and N6-isopentenyladenosine (iPR), generally showed decreasing trends within the degeneration window (Figure 3A). These results indicate that inflorescence degeneration was accompanied by marked changes in hormone profiles.

To further identify key hormones more closely associated with the degeneration window, statistical tests were performed on the 19 hormones described above (Table S1). The results showed that ABA, JA, MeJA, and cZR differed significantly in the degeneration window compared with the adjacent normally developing inflorescences on both sides of the developmental axis, namely ZH2 and ZH5 (Figure 3B–E). Specifically, ABA levels in BY3 were increased by 30.0- and 25.0-fold compared with ZH2 and ZH5, respectively, while those in BY4 were increased by 25.0- and 15.0-fold, respectively (Figure 3B). JA levels in BY3 were increased by approximately 3.0- and 3.1-fold compared with ZH2 and ZH5, respectively, while those in BY4 were increased by 3.6- and 3.8-fold, respectively (Figure 3D). MeJA levels in BY3 were increased by 2.8- and 7.4-fold compared with ZH2 and ZH5, respectively, while those in BY4 were increased by 1.4- and 5.1-fold, respectively (Figure 3E). In contrast, cZR levels in BY3 were 11.7- and 9.4-fold lower than those in ZH2 and ZH5, respectively, while those in BY4 were 2.8- and 2.3-fold lower, respectively (Figure 3C).

Notably, these four hormones did not exhibit significant differences of comparable magnitude among normally developing inflorescence stages (Figure 3B–E), further supporting the window-specific nature of their changes during degeneration. By comparison, IAA and some GA- and tZ-related hormones also differed significantly between degenerated and adjacent inflorescences, but they also fluctuated among adjacent normally developing inflorescences (Figure 3F–H). Taken together, the degeneration window of areca palm inflorescences exhibited pronounced remodeling of hormone profiles, with increased ABA, JA, and MeJA levels and decreased cZR levels representing the most prominent hormonal features of this stage. These findings provide a clear direction for subsequent transcriptome-based analyses of hormone signaling pathways and key nodal genes.

### 2.4. Characteristics of Differentially Expressed Genes Within the Degeneration Window

**Figure 4:**
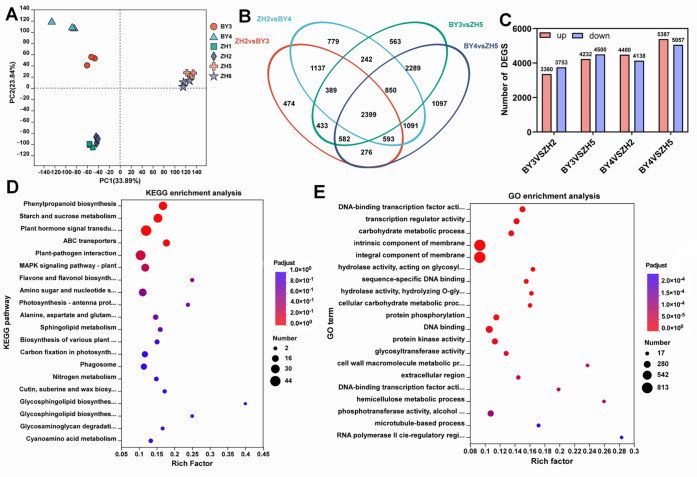
Transcriptomic differential expression characteristics within the degeneration window. (**A**) Principal component analysis of transcriptome data from inflorescence samples at different developmental stages. (**B**) Intersection analysis of differentially expressed genes identified from the four comparisons: BY3 vs. ZH2, BY3 vs. ZH5, BY4 vs. ZH2, and BY4 vs. ZH5. (**C**) Statistical summary of the number of differentially expressed genes (DEGs) in comparisons between the degeneration window and adjacent normally developing stages. (**D**,**E**) KEGG pathway enrichment analysis (**D**) and GO enrichment analysis (**E**) of the shared differentially expressed genes across the four comparisons. The top 20 enriched pathways or terms are shown. Bubble size represents the number of genes enriched in each corresponding pathway or term, and color indicates the adjusted significance level.

To characterize the molecular expression features associated with the window of areca palm inflorescence degeneration, RNA-seq analysis was performed on inflorescences at different developmental stages. High-quality clean reads were obtained from all samples, successfully mapped to the areca palm reference genome, and used for expression quantification. The biological replicates showed good consistency (Table S2; Figure S3). Principal component analysis (PCA) showed that PC1 and PC2 explained 45.97% and 28.42% of the total variance, respectively, with a cumulative explained variance of 74.39% (Figure 4A). Biological replicates from each stage clustered closely, and clear separation was observed among different stages. Notably, the degeneration-window stages, BY3 and BY4, were clearly separated from the adjacent normally developing stages, ZH5 and ZH2, in the PCA space (Figure 4A), indicating that their transcriptomic expression profiles differed markedly from those of normally developing inflorescences.

Differential expression analysis was further performed between the degeneration-window stages and adjacent normally developing stages (padj < 0.05, |log_2_FC| ≥ 2). The results showed that 3360 genes were upregulated and 3753 genes were downregulated in BY3 vs. ZH2; 4232 genes were upregulated and 4500 genes were downregulated in BY3 vs. ZH5; 4480 genes were upregulated and 4138 genes were downregulated in BY4 vs. ZH2; and 5387 genes were upregulated and 5057 genes were downregulated in BY4 vs. ZH5 (Figure 4C). These results indicate extensive transcriptional reprogramming in the degeneration window compared with the adjacent normally developing inflorescences. To identify molecular signals more representative of the inflorescence degeneration window, BY3 and BY4, the differentially expressed genes from the four comparisons were further subjected to intersection analysis. A total of 2399 shared differentially expressed genes were identified across the four comparisons, constituting a stable core expression-response gene set within the degeneration window (Figure 4B). Functional enrichment analysis of this shared gene set showed that KEGG pathways were significantly enriched in phenylpropanoid biosynthesis, starch and sucrose metabolism, and plant hormone signal transduction (Figure 4D), whereas GO enrichment mainly involved DNA-binding transcription factor activity, transcription regulator activity, and carbohydrate metabolism-related processes (Figure 4E).

### 2.5. Expression Patterns of Genes Involved in Starch and Sucrose Metabolism and Plant Hormone Signal Transduction Within the Degeneration Window

**Figure 5:**
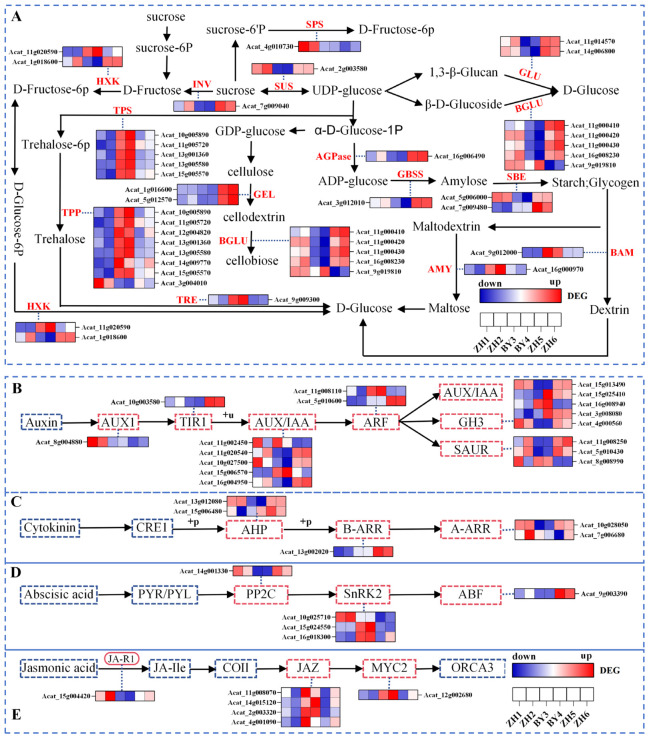
Key differentially expressed genes involved in starch and sucrose metabolism and plant hormone signal transduction pathways. (**A**) Differentially expressed genes involved in the starch and sucrose metabolism pathway. (**B**–**E**) Differentially expressed genes involved in plant hormone signal transduction pathways, including the auxin (IAA) signaling pathway (**B**), cytokinin (CK) signaling pathway (**C**), abscisic acid (ABA) signaling pathway (**D**), and jasmonic acid (JA) signaling pathway (**E**). Expression levels are shown after log_2_(TPM + 1) transformation. The main abbreviations in the figure are as follows: SPS, sucrose-phosphate synthase; SUS, sucrose synthase; INV, invertase; TPS, trehalose-6-phosphate synthase; TPP, trehalose-6-phosphate phosphatase; TRE, trehalase; HXK, hexokinase; AGPase, ADP-glucose pyrophosphorylase; GBSS, granule-bound starch synthase; SBE, starch branching enzyme; AMY, α-amylase; BAM, β-amylase; β-amylase; GLU, 1,3-β-glucanase; BGLU, β-glucosidase; GEL, endoglucanase;AHP, histidine phosphotransfer protein; A-ARR, type-A response regulator; PP2C, protein phosphatase 2C; SnRK2, SNF1-related protein kinase 2; ABF, ABA-responsive element-binding factor; JAR1, jasmonate resistant 1; JAZ, jasmonate ZIM-domain protein; MYC2, transcription factor MYC2; AUX1, auxin influx carrier 1; TIR1, transport inhibitor response 1; AUX/IAA, transcriptional repressor; ARF, auxin response factor; GH3, GH3 family protein; SAUR, small auxin-up RNA; CRE1, cytokinin response 1; B-ARR, type-B response regulator; PYR/PYL, pyrabactin resistance/pyrabactin resistance-like; JA-Ile, jasmonoyl-L-isoleucine; COI1, coronatine insensitive 1; ORCA3, AP2/ERF transcription factor ORCA3; DEG, differentially expressed gene; +u and +p indicate ubiquitination and phosphorylation, respectively.

KEGG enrichment analysis showed that the shared differentially expressed genes associated with the degeneration window were mainly enriched in the starch and sucrose metabolism pathway and the plant hormone signal transduction pathway. The standardized heatmap further showed that, during the degeneration-window stages (BY3 and BY4), multiple key nodal genes in these pathways exhibited directional expression changes (Figure 5). In the starch and sucrose metabolism pathway, a total of 31 DEGs were identified. Regarding sucrose metabolism, the sucrose synthase (SUS)-encoding gene *AcSUS2* (Acat_2g003580) and the invertase (INV)-encoding gene *AcINV3* (Acat_7g009040) were both downregulated within the degeneration window. By contrast, although the sucrose-phosphate synthase (SPS)-encoding gene *AcSPS4* (Acat_4g010730) showed altered expression, it did not display a downregulation pattern consistent with those of the former two genes (Figure 5A). Regarding starch metabolism, key genes involved in starch synthesis, including the ADP-glucose pyrophosphorylase (AGPase)-encoding gene *AcAPL2* (Acat_16g006490), the granule-bound starch synthase (GBSS)-encoding gene *AcWAXY* (Acat_3g012010), and the starch branching enzyme (SBE)-encoding gene *AcSBE1* (Acat_5g006000), were all downregulated within the degeneration window, suggesting that starch synthesis-related processes were inhibited. In contrast, genes associated with starch degradation generally showed an upregulation trend. Among them, the α-amylase (AMY)-encoding gene *AcAMY* (Acat_16g000970) and the β-amylase (BAM)-encoding gene *AcBAM1* (Acat_9g012000) were upregulated within the degeneration window (Figure 5A). Given that β-amylase participates in starch degradation and soluble sugar release, the upregulation of *AcBAM1* further supports the marked enhancement of starch degradation during the degeneration window. In addition to sucrose and starch metabolism, genes associated with the trehalose metabolism branch also showed an overall upregulation trend within the degeneration window. Specifically, trehalose-6-phosphate synthase (TPS)-encoding genes were generally upregulated; the trehalose-6-phosphate phosphatase (TPP)-encoding genes *AcTPPG* (Acat_12g004820) and *AcTPP6* (Acat_14g009770), as well as the trehalase (TRE)-encoding gene *AcTRE* (Acat_9g009300), were all upregulated within the degeneration window (Figure 5A).

In the plant hormone signal transduction pathway, a total of 44 DEGs were identified. In the auxin (IAA) signaling pathway, the auxin receptor TIR1-encoding gene *AcTIR1* (Acat_10g003580) was significantly downregulated within the degeneration window. Meanwhile, members of the *AUX/IAA, GH3* and *SAUR* families also showed differential expression. Among them, *AcARF1* (Acat_11g008110) was upregulated, whereas *AcIAA2* (Acat_11g020540), *AcIAA3* (Acat_10g027500) and *AcIAA5* (Acat_16g004950) were downregulated (Figure 5B). These results indicate that auxin signal perception and downstream response processes were markedly altered within the degeneration window. In the cytokinin (CK) signaling pathway, two key genes were both downregulated within the degeneration window, namely the histidine phosphotransfer protein gene *AcAHP2* (Acat_13g012080) and the type-A response regulator gene *AcORR3* (Acat_10g028050) (Figure 5C). These two genes are located in the cytokinin phosphorelay and response output modules, respectively, suggesting that both CK signal transduction and response were suppressed within the degeneration window. In the abscisic acid (ABA) signaling pathway, four key genes exhibited directional changes. The PP2C-encoding gene *AcPP2C50* (Acat_14g001330) and the ABF-encoding gene *AcABF* (Acat_9g003390) were both downregulated within the degeneration window, whereas the SnRK2-encoding genes *AcSnRK2* (Acat_15g024550) and *AcSnRK2-10* (Acat_16g018300) were upregulated (Figure 5D), indicating directional expression changes in key nodal genes of the ABA signaling pathway. In the jasmonic acid (JA) signaling pathway, the jasmonate resistant 1 gene *AcJAR1* (Acat_15g004420) was downregulated within the degeneration window, whereas the JAZ-encoding gene *AcJAZ* (Acat_2g003320) and the transcription factor *AcMYC2* (Acat_12g002680) were upregulated (Figure 5E). MYC2 is an important transcriptional regulatory node in JA signaling, and its upregulation indicates that the response module of this pathway was altered in degenerated inflorescences.

### 2.6. WGCNA Identification of Co-Expression Modules and Hub Genes

**Figure 6:**
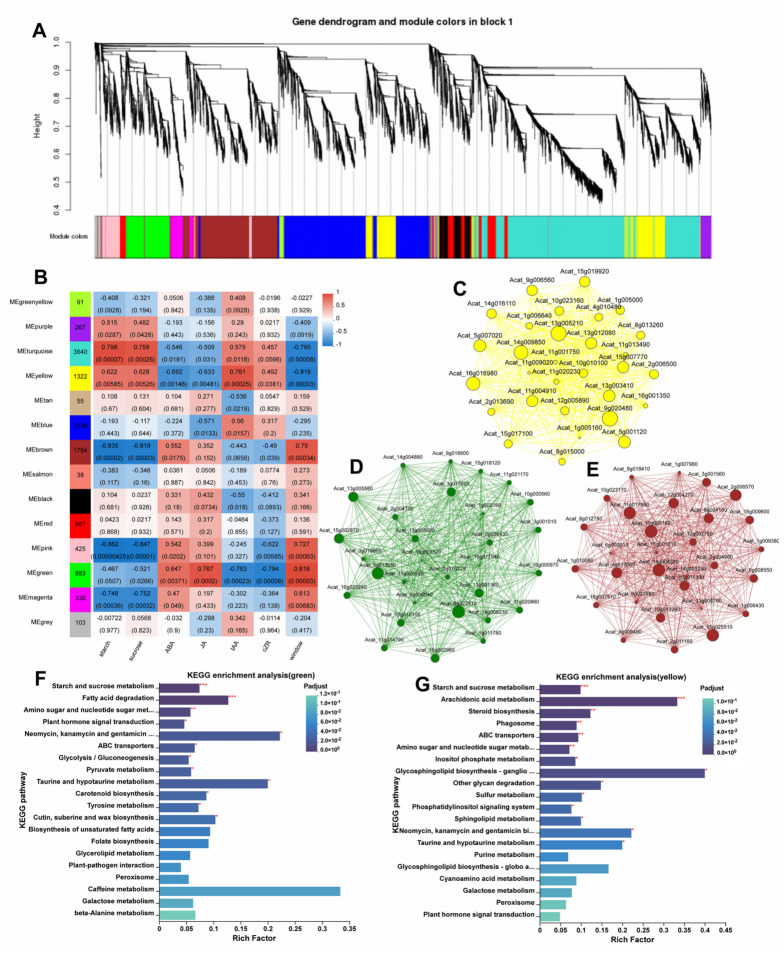
Weighted gene co-expression network analysis of areca palm inflorescence degeneration. (**A**) Gene co-expression clustering dendrogram and module assignment. Different colors represent different co-expression modules, and each branch represents an individual gene. (**B**) Heatmap of module–trait correlations. The *x*-axis represents different physiological and morphological traits, and the *y*-axis represents module eigengenes. (**C**–**E**) Co-expression networks constructed from the top 30 genes with the highest connectivity in the yellow, green, and brown modules, corresponding to (**C**), (**D**), and (**E**), respectively. Node size is proportional to gene connectivity, and edges indicate co-expression relationships between genes. (**F**,**G**) KEGG enrichment analysis of the green module (**F**) and yellow module (**G**). Color indicates the adjusted significance level (Padjust). Asterisks in (**F**,**G**) indicate significance levels: * Padjust < 0.05, ** Padjust < 0.01, and *** Padjust < 0.001.

To elucidate the molecular basis of areca palm inflorescence degeneration and identify key hub genes, WGCNA was performed based on the transcriptomic expression matrix. The original matrix contained 31,179 genes. After filtering by expression level and variation, 12,535 genes were retained for network construction (signed, Sβ = 20), and 14 co-expression modules were identified (Figure 6A). Among these modules, the yellow, green, and brown modules showed strong correlations with sucrose, starch, IAA, JA, cZ, ABA, and the degeneration-window phenotype (Figure 6B). KEGG enrichment analysis showed that the green module was mainly enriched in starch and sucrose metabolism and plant hormone signal transduction, whereas the yellow module was enriched in the starch and sucrose metabolism pathway (Figure 6F,G), indicating that the modules associated with inflorescence degeneration were functionally related to carbon metabolism and hormone regulation. Based on intramodular connectivity, six key hub genes were further screened from the top 30 highly connected genes in the yellow, green, and brown modules, namely *AcAHP2* (Acat_13g012080), *AcTIFY4B* (Acat_11g001750), *AcTPS9-2* (Acat_13g001360), *AcHXK2* (Acat_11g020590), *AcWRKY3* (Acat_14g006230), and *AcMPK1* (Acat_14g003280).

### 2.7. qRT-PCR Validation of Selected Gene Expression

**Figure 7:**
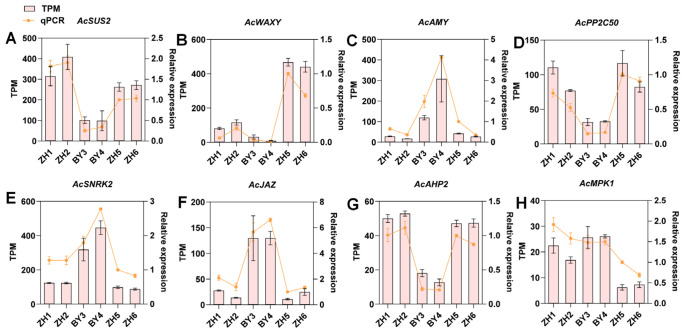
qRT-PCR validation of the expression patterns of candidate genes in inflorescences at different developmental stages. (**A**–**H**) Expression changes of *AcSUS2*, *AcWAXY*, *AcAMY*, *AcPP2C50, AcSnRK2*, *AcJAZ*, *AcAHP2,* and *AcMPK1*, respectively. Bars represent TPM values obtained from RNA-seq data (left *y*-axis), and lines represent relative expression levels determined by qRT-PCR (right *y*-axis). The expression trends detected by qRT-PCR were generally consistent with those obtained from RNA-seq, confirming the reliability of the transcriptome data.

To validate the reliability of the transcriptome data, eight genes involved in starch and sucrose metabolism or plant hormone signaling pathways or identified as hub genes by WGCNA were selected for qRT-PCR validation. The starch and sucrose metabolism-related genes *AcSUS2* and *AcWAXY* were downregulated within the degeneration window (Figure 7A,B), whereas *AcAMY* was upregulated (Figure 7C). In the hormone signaling pathways, the ABA pathway-related genes *AcPP2C50* and *AcSnRK2* showed opposite expression patterns within the degeneration window, with *AcPP2C50* being downregulated and *AcSnRK2* being upregulated (Figure 7D,E), and the JA-related gene *AcJAZ* was upregulated in degenerated inflorescences (Figure 7F). Among the hub genes identified by WGCNA, *AcAHP2* and *AcMPK1* showed decreasing and increasing trends, respectively, within the degeneration window (Figure 7G,H). The expression trends of all eight genes were consistent with the RNA-seq results.

## 3. Discussion

### 3.1. Inflorescence Degeneration in Areca Palm Occurs Within a Specific Developmental Window

Inflorescence degeneration and reproductive organ abortion are common biological phenomena during reproductive development in perennial woody crops. Previous studies have shown that abnormal development of reproductive organs in most crops exhibits clear developmental-window specificity rather than occurring randomly and is often concentrated at specific developmental stages that are highly sensitive to environmental conditions, nutrient supply, and endogenous regulatory signals [29,30]. For example, floret abortion in wheat mainly occurs at pre-anthesis stages [31], and developing inflorescences of litchi can shift from normal elongation to shrinkage under high-temperature conditions [32]. Similarly, studies on floral bud differentiation in tomato have shown that abnormal inflorescence branching is restricted to the first 48 h after floral primordium initiation [33]. In perennial fruit trees such as citrus, drought stress during floral bud differentiation specifically causes a sharp reduction in flower number in the following year, rather than merely delaying flowering time [34]. Collectively, these findings suggest that reproductive organs are more sensitive to internal and external regulatory disturbances during specific developmental periods. It should be noted that although genetic and environmental interaction models have been reported for the developmental windows of reproductive organ abortion in gramineous crops (e.g., rice panicle degeneration) and in the tropical woody crop oil palm (in which the major abortion stage occurs approximately 10 months before harvest) [8,35], physiological and molecular dissection of the sensitive period for inflorescence degeneration remains lacking in areca palm, an important tropical economic crop. The present study showed that inflorescence degeneration in areca palm also exhibits such a developmental-window pattern, with degeneration mainly concentrated at the BY3 and BY4 stages during the full-bloom stage. At these stages, degeneration phenotypes, including reduced tissue size, blackening, and growth arrest, were concentrated and accompanied by coordinated changes in carbohydrate contents, hormone profiles, and transcriptomic status. This indicates that these stages are not merely morphological transition stages, but more likely represent a critical window during which normally developing inflorescences shift toward degeneration.

### 3.2. Restricted Carbon Supply and Shifts in Carbon Metabolic Status Are Involved in Areca Palm Inflorescence Degeneration

Reproductive organ development is highly dependent on assimilate input and sink stability during critical periods [36]. Therefore, flower or floral bud degeneration and abortion often reflect an imbalance at the level of carbon supply [37]. Recent studies have shown that abnormal carbohydrate allocation may directly determine reproductive organ fate. Once developing floral buds or grains enter a weak-sink state, their growth can be inhibited by unfavorable carbon allocation. For example, in Anthurium, carbon competition between young leaves and simultaneously developing floral buds can inhibit continued floral bud growth and lead to abortion [15]. In peanut, weight reduction during critical developmental stages is closely associated with decreased sucrose import and conversion capacity [38]. The phenomena observed in the BY3/BY4 degeneration window of areca palm inflorescences in this study are highly consistent with these findings. Degenerated inflorescences not only exhibited weight-reduction characteristics such as reduced size and stagnation of dry matter accumulation, but also showed a significant decrease in sucrose content, indicating that the metabolic activity of the inflorescence as a carbon sink had substantially declined at this stage. Thus, restricted carbon supply is not simply attributable to reduced export from source leaves but is more likely due to the loss of sucrose unloading and conversion capacity within the inflorescence itself. This functional imbalance at the level of carbon metabolism ultimately triggers the transition of inflorescences from normal development to irreversible degeneration.

Transcriptome analysis further revealed that abnormal sugar metabolism within the degeneration window was not merely a simple “decline in reserves” but was accompanied by a directional shift in carbon metabolic status. The starch synthesis-related gene *AcAPL2* was downregulated in degenerated inflorescences (Figure 5A). This gene encodes an enzyme involved in a key initial step of starch biosynthesis. In tomato, overexpression of the homologous gene *VaAPL1* enhances starch synthesis and continuously supports soluble sugar accumulation, indicating that this type of gene is closely associated with the establishment of local carbon reserves and the maintenance of sink strength [39]. In addition, the starch degradation gene *AcBAM1* was upregulated in degenerated inflorescences (Figure 5A). Its homologous gene *BAM1* participates in starch degradation and carbon availability regulation in Arabidopsis, especially under stress conditions, where it can promote starch mobilization to maintain the supply of sucrose and downstream metabolites [40]. Meanwhile, the sucrose metabolism gene *AcSUS2* was downregulated within the degeneration window (Figure 5A). *SUS* genes are generally regarded as important nodes connecting sucrose cleavage with subsequent carbon utilization in sink tissues, and their reduced expression indicates that degenerated inflorescences not only experienced insufficient assimilate input, but also had reduced capacity for effective sugar utilization and conversion [41]. Similar phenomena have been widely reported in other crops. After downregulation of *CsSUS4* in cucumber, the contents of hexose and starch in flowers and fruits decrease, and floral organ size and weight are both inhibited [42]. In cotton, reduced *SuS* activity is closely associated with increased seed abortion, whereas overexpression of *SuS* can effectively reduce seed abortion [43]. In this study, the marked downregulation of *SuS* gene expression observed at the BY3/BY4 stages of degenerated areca palm inflorescences indicates that the carbon metabolic function of degenerated inflorescences had substantially declined at core nodes of sink tissues. In addition, changes in the trehalose metabolism branch suggest that upstream regulatory resetting may occur within the degeneration window. *AcTPS9-2* is a member of the class II TPS family, and the Tre6P/TPS–TPP module is closely associated with sugar-status sensing and carbon allocation, with class II TPS proteins considered important regulatory factors in this process [44]. At the BY3/BY4 stages, the overall increase in the expression of *AcTPS9-2* and other trehalose pathway genes (Figure 5A), together with the significant decrease in trehalose content, suggests that the sugar-sensing system of degenerated inflorescences may be adjusted, shifting their carbon utilization mode from supporting sustained development toward a more conservative response state.

### 3.3. Hormonal Network Remodeling Further Modulates Inflorescence Growth Within the Degeneration Window

Plant hormones do not regulate inflorescence development by acting on a single metabolic process alone; rather, they jointly determine the developmental status of inflorescences through coordinated regulation of cell division, tissue expansion, and subsequent organ formation [45,46]. When hormonal balance is disrupted at different developmental stages, inflorescence development may shift from sustained growth to stagnation, shrinkage, and even degeneration [47]. In abortive maize kernels under shading, the contents of IAA, GA, and ZR decrease simultaneously, whereas ABA continues to accumulate [48]; cytokinin contents also decrease sharply under stress conditions [49]. These examples indicate that attenuation of growth-promoting hormones and accumulation of inhibitory hormones are common endocrine features of reproductive organ abortion. In this study, IAA and cZR contents decreased at the BY3/BY4 stages, whereas ABA, JA, and MeJA contents increased (Figure 3B–F), which is consistent with the above pattern. Auxin and cytokinin are two major hormonal systems supporting sustained inflorescence formation: the former acts as a key morphogenetic signal for floral primordium initiation and organ polarity establishment, whereas the latter maintains meristem activity and promotes cell division to ensure continuous organ initiation [45,50]. However, at the BY3/BY4 stages within the degeneration window, both IAA and cZR contents showed marked decreasing trends (Figure 3), and their signaling pathways were coordinately weakened at multiple levels. At the level of auxin perception, the TIR1 receptor-encoding gene *AcTIR1* was downregulated (Figure 5B). TIR1-mediated degradation of Aux/IAA proteins is a key molecular switch that initiates auxin transcriptional responses, and its downregulation suggests that the ability of inflorescences to perceive and respond to auxin signals was weakened. Previous studies have shown that normal expression of *TIR1* homologs in tomato is required for the successful transition from flower to fruit [51]. Meanwhile, *AcAHP2* and *AcORR3* in the cytokinin signaling pathway also showed persistently low expression levels (Figure 5C), indicating that the overall cytokinin signal transduction chain was impaired. Therefore, at the BY3/BY4 stages, degenerated inflorescences not only lacked sufficient auxin and cytokinin signals but also lost the capacity to effectively perceive and transduce these signals. The molecular driving forces required to maintain organ initiation, cell division, and tissue expansion were therefore constrained at the hormonal level.

JA and its active derivatives are important signaling molecules regulating plant reproductive organ development and are also core factors mediating the growth–defense balance [52,53]. Activation of JA signaling usually inhibits vegetative growth and preferentially initiates stress-defense programs, inducing ROS accumulation, autophagy, and even local cell death, which may further lead to inflorescence growth arrest, shrinkage, and even abortion [54,55]. In this study, JA and MeJA contents were significantly increased during the inflorescence degeneration window; however, the gene involved in JA activation, *AcJAR1*, showed a downward expression trend, whereas the core transcription factor *AcMYC2* remained highly expressed (Figure 5E). As a key upstream enzyme in the jasmonate pathway, JAR1 catalyzes the conversion of JA into its active form, jasmonoyl-L-isoleucine (JA-Ile) [56]. MYC2 functions at the downstream execution layer of the pathway and serves as a core regulatory node mediating JA signal output [57]. Previous studies have confirmed that JAR1 determines the efficiency of JA-Ile biosynthesis, whereas MYC2 can drive JA-dependent transcriptional reprogramming and directly regulate meristem activity and growth inhibition processes [58]. Taken together, these results suggest that the jasmonate pathway in the degeneration window is not activated in a continuous linear manner from upstream to downstream but instead undergoes selective reorganization and amplification of signaling modules. The sustained high expression of *AcMYC2* suggests that the regulatory focus of inflorescence development may have shifted from organ formation toward stress responses and resource redistribution, thereby promoting the progression of inflorescence degeneration.

In summary, areca palm inflorescence degeneration is not independently regulated by a single hormonal pathway but is the result from coordinated interactions among multiple hormones. Under conditions of carbon metabolic imbalance and restricted carbon supply, the IAA- and CK-mediated growth regulatory systems are weakened, thereby impairing normal inflorescence development; enhanced ABA signaling accelerates growth arrest and physiological decline; and remodeling of the jasmonate pathway further activates stress responses and promotes redistribution of endogenous resources. The coupling of multiple hormonal signals disrupts the dynamic balance between inflorescence growth and homeostasis maintenance, inhibits tissue proliferation and organ formation, and ultimately leads to inflorescence shrinkage and subsequent degeneration.

### 3.4. Co-Expression Network Reveals a Potential Mechanism by Which Sugar Signaling and Hormones Coordinately Drive Inflorescence Degeneration

**Figure 8:**
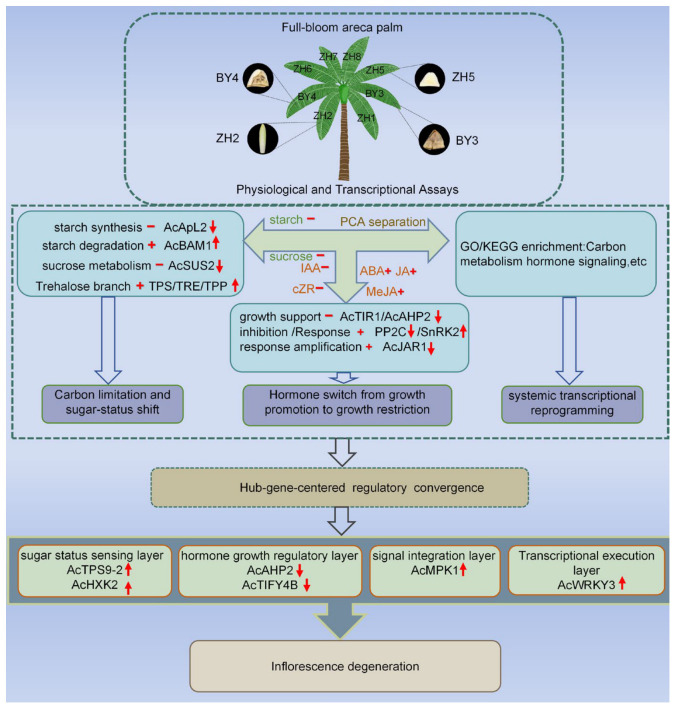
Hypothetical working model of inflorescence degeneration in areca palm. Carbon metabolic imbalance, hormonal network remodeling, and transcriptional reprogramming act together within the degeneration window (BY3/BY4), converge at the level of key hub genes, and ultimately promote inflorescence degeneration. Arrows indicate changes in gene expression within the degeneration window; plus and minus signs indicate the enhancement or attenuation of related metabolites or processes.

Based on the global transcriptional reprogramming observed within the degeneration window, WGCNA further narrowed these large-scale expression changes to a limited number of key modules and hub genes. The six core hub genes identified in this study are involved in multiple key processes, including sugar metabolism and sugar signal perception (*AcTPS9-2* and *AcHXK2*), hormone signaling regulation (*AcAHP2* and *AcTIFY4B*), signal transduction (*AcMPK1*), and transcriptional regulation (*AcWRKY3*). In this study, sucrose and starch contents were significantly decreased in BY3 and BY4 inflorescences, whereas the expression levels of the key sugar-signaling genes *AcTPS9-2* and *AcHXK2* were markedly upregulated (Figure 8). This pattern, in which carbohydrate reserves declined while sugar-sensing pathways were activated, indicates that the inflorescences were not in a carbon-sufficient state, but instead exhibited a specific sensing and responses to intracellular sugar metabolic imbalance. In Arabidopsis, loss of *TPS1* directly leads to embryo abortion [59]. More directly, studies in lotus have shown that upregulation of *TPS1* significantly reduces the rate of low-light-induced floral bud abortion [60]. Evidence from these different species collectively suggests that sugar homeostasis and signal perception mediated by *TPS* family genes may represent a conserved regulatory node determining reproductive organ fate. Similarly, as a core component of sugar signal perception and metabolism, HXK dysfunction is closely associated with reproductive developmental failure. In tomato, silencing *SlHXK1* not only increases flower abscission and reduces pollen fertility but also disrupts auxin and ethylene signaling pathways [61]. In rice, suppression of *OsHXK10* similarly leads to impaired anther dehiscence and pollen germination [62]. Therefore, the increased expression of *AcTPS9-2* and *AcHXK2* may reflect a compensatory sensing response to sugar deprivation in the inflorescences. However, under conditions in which carbon supply can no longer support inflorescence formation, sustained activation of this signal may fail to restore metabolic homeostasis and may instead reinforce downstream growth inhibition and stress-response programs, thereby accelerating the irreversible progression of degeneration.

Following the abnormal sugar status, hormonal imbalance may represent a key step driving inflorescences toward degeneration. In degenerated inflorescences, *AcAHP2*, a core nodal gene involved in cytokinin signal transduction, was downregulated (Figure 8) [63]. This was consistent with the decrease in cZR content, together indicating that the supporting capacity of CK signaling for sustained inflorescence differentiation and expansion was weakened. Meanwhile, *AcTIFY4B*, a member of the *TIFY* family widely involved in organ size regulation and hormone responses, was also downregulated (Figure 8). Given that *TIFY4B/PPD*-type members have been shown in multiple crops to be closely associated with organ growth vigor [64], its reduced expression further suggests that the regulatory interface maintaining normal inflorescence expansion had been weakened. Together with the decreases in IAA and cZR levels and the enhancement of ABA- and JA-related responses, these changes indicate that, under the stress of carbon metabolic imbalance, the hormonal regulatory focus within inflorescences had shifted from “maintaining organ formation” toward “restricting growth and responding to stress”. Studies in wheat provide a clear model for this process: insufficient sucrose supply induces ABA and JA biosynthesis, leading to reduced fertility of basal spikelets [65].

At the levels of signal integration and transcriptional execution, *AcMPK1* and *AcWRKY3* both showed high expression at the BY3/BY4 stages (Figure 8), suggesting that they may contribute to the amplification and stabilization of upstream disturbances, respectively. MAPK cascades can amplify metabolic and hormonal disturbances into sustained cellular responses, as observed in ovule abortion in Xanthoceras sorbifolium, where activation of the MAPK pathway transmits metabolic signals to downstream cell death programs [66]. WRKY transcription factors are located at the intersection of developmental and stress regulation. In rapeseed, *BnaWGR1* converts transient stress signals into an irreversible senescence phenotype by inducing ROS accumulation [67]. As one of the few overlapping nodes between shared differentially expressed transcription factors and core genes of key modules, *AcWRKY3* may integrate upstream metabolic–hormonal imbalance into stable transcriptional outputs. Accordingly, *AcMPK1* is proposed to mainly function in signal relay and cascade amplification, whereas *AcWRKY3* may execute downstream transcriptional regulation. The coordinated action of these two genes may further convert early physiological disturbances into sustained transcriptional regulatory changes, thereby promoting the progression of inflorescence degeneration.

Taken together, this study proposes a hypothetical working model for areca palm inflorescence degeneration (Figure 8). Within the critical BY3/BY4 window, restricted carbon supply and abnormal sugar-status perception, reflected by decreases in sucrose and starch contents, first emerge and are manifested at the sugar-signaling layer represented by *AcTPS9-2* and *AcHXK2*. Subsequently, weakened IAA and cZR support and enhanced ABA- and JA-related responses, together with imbalance at the hormone–growth regulatory interface represented by *AcAHP2* and *AcTIFY4B*, shift the organ from a normal formation state toward a physiologically inhibitory and stress-biased state. On this basis, *AcMPK1* further integrates and amplifies these upstream signals, which are ultimately stabilized into a sustained degeneration program by transcriptional regulatory nodes such as *AcWRKY3*, thereby promoting the transition of inflorescences from normal formation to degeneration. It should be noted that this mechanism remains a hypothetical model proposed based on the integration of physiological indicators, expression patterns, and co-expression networks, and still requires further validation through temporal expression analysis and functional studies. From the perspective of practical application, the findings of this study may provide useful references for stabilizing areca palm yield and supporting future yield improvement at multiple levels. First, the identification of BY3/BY4 as a high-incidence degeneration window provides a specific developmental stage for the early diagnosis and targeted monitoring of inflorescence degeneration in the field, which may help guide focused observation and management before irreversible degeneration occurs. Second, the significant decreases in sucrose, starch, trehalose, and cZR levels, together with the significant increases in ABA, JA, and MeJA levels within the degeneration window, suggest that these physiological indicators may serve as candidate indicators for evaluating the degree of inflorescence degeneration and the effectiveness of cultivation and management practices. Third, the six hub genes identified by WGCNA may provide candidate targets for screening degeneration-resistant germplasm, developing early molecular markers, and supporting future molecular breeding. Therefore, although further field validation and functional studies are still required, these results provide a physiological and molecular basis for reducing areca palm inflorescence degeneration, maintaining effective inflorescence formation, and improving yield stability.

## 4. Materials and Methods

### 4.1. Plant Materials and Sample Collection

This study was conducted in 2025 in Qiongzhong Li and Miao Autonomous County, Hainan Province, China. Healthy mature areca palm plants with uniform field management and normal growth were selected as the experimental materials. In areca palm, inflorescence primordia emerge synchronously with leaves, and each leaf axil encloses one inflorescence primordium. Because their developmental status cannot be directly determined from external morphology, dissection is required for accurate identification (Figure S1). Samples collected at the full-bloom stage in June were selected for subsequent experiments. Inflorescence primordia corresponding to consecutive leaf positions were further dissected from six plants at the full-bloom stage. Among them, five plants showed a relatively consistent pattern of degeneration distribution and were therefore selected for formal sampling. At the sampling time point, inflorescence primordia from eight consecutive leaf positions were collected from the outer to the inner leaves according to leaf order and were sequentially designated as ZH1, ZH2, BY3, BY4, ZH5, ZH6, ZH7, and ZH8. Among these, BY3 and BY4 represented the leaf positions with a high incidence of degeneration, whereas the remaining positions represented normally developing inflorescences (Figure 1A). For each leaf position, equal amounts of material were collected from the five plants and pooled to generate three replicate samples. After collection, the samples were rapidly aliquoted, frozen in liquid nitrogen, and stored at −80 °C for plant hormone determination, carbohydrate measurement, and RNA extraction. For subsequent plant hormone determination and transcriptome sequencing, six representative stages, namely ZH1, ZH2, BY3, BY4, ZH5, and ZH6, were selected for analysis. Among them, ZH2 and ZH5 were used as the nearest normally developing inflorescences on both sides of the degeneration window for focused comparison.

### 4.2. Measurement of Inflorescence Morphological Traits

To characterize morphological changes in inflorescences at different leaf positions, the length, width, and fresh weight of inflorescences from eight consecutive leaf positions of five experimental plants were measured. For length and width measurements, ZH1 and ZH2 inflorescences were relatively large; therefore, a steel ruler was used to measure their length (the maximum distance from the base to the apex of the inflorescence) and width (the transverse distance at the widest part of the inflorescence). Inflorescences at the remaining leaf positions were smaller and were measured using a digital vernier caliper (0–150 mm, resolution 0.01 mm; Shanghai Shengong, Shanghai, China). For fresh weight determination, ZH1 and ZH2 inflorescences were weighed using an electronic bench scale (model ALH-3, measuring range 0–3000 g, readability 0.01 g; Shanghai Yingzhan, Shanghai, China), whereas inflorescences at the remaining leaf positions were weighed using an analytical balance (model FA2004, readability 0.0001 g; Shanghai Yueping, Shanghai, China). Before weighing, surface moisture was gently removed from all samples using filter paper.

### 4.3. Determination of Endogenous Hormone Contents in Inflorescences

Endogenous hormones and related metabolites in inflorescences were determined by liquid chromatography–electrospray ionization tandem mass spectrometry (LC-ESI-MS/MS) [68]. Briefly, 100 mg of each inflorescence sample was accurately weighed and placed in a 2 mL grinding tube, followed by the addition of 498 μL of 80% methanol extraction solvent and 2 μL of salicylic acid-d4 (SA-d4) internal standard solution (2 μg/mL). After mixing, the samples were ground at low temperature for 3 min using a cryogenic grinder (Wonbio-96E; Shanghai Wanbo Biotechnology Co., Ltd., Shanghai, China) and ultrasonically extracted at low temperature for 1 h using an ultrasonic cleaner (SBL-10DT; Ningbo Scientz Biotechnology Co., Ltd., Ningbo, China). The extract was then mixed with an EN15662 extraction salt packet, vigorously shaken for 10 min, and centrifuged at 10 °C for 10 min. The supernatant was diluted for LC–MS/MS analysis. Chromatographic separation was performed on a Waters BEH C18 column (2.1 mm × 100 mm, 1.7 μm; Waters Corporation, Milford, MA, USA), using 0.1% formic acid in water and 0.1% formic acid in acetonitrile as mobile phases for gradient elution, with a column temperature of 30 °C, an injection volume of 10 μL, and a flow rate of 0.35 mL/min. Mass spectrometric detection was performed using an AB SCIEX QTRAP 6500+ triple quadrupole/linear ion trap mass spectrometer (SCIEX, Framingham, MA, USA), and data were acquired in Scheduled MRM mode under positive/negative ion-switching conditions. Quantification was performed using the internal standard method. Standard curves were established based on the peak-area ratios of the target analytes to the internal standard, and hormone contents were calculated according to sample weight.

### 4.4. Determination of Carbohydrate Contents in Inflorescences

Sucrose and starch contents were measured using commercial assay kits from Beijing Solarbio Science & Technology Co., Ltd. (Beijing, China), with catalog numbers BC2460 (Sucrose Assay Kit) and BC0700 (Starch Assay Kit), respectively. Trehalose content was determined using a commercial trehalose assay kit from Jiangsu Addison Biotechnology Co., Ltd. (Suzhou, China), with catalog number ADS-W-TDX006. For all samples, reagent proportions, reaction conditions, and operating procedures were performed strictly according to the manufacturers’ instructions to ensure standardization of the experimental system and reproducibility of the results. Sucrose content was determined using the resorcinol colorimetric method. Under acidic conditions, sucrose is hydrolyzed into glucose and fructose, and the released fructose further reacts with resorcinol to form a colored product, whose absorbance was measured at 480 nm. Starch content was determined using the anthrone colorimetric method. Briefly, soluble sugars were first removed from the samples with 80% ethanol to separate starch from soluble sugars. The remaining starch was then hydrolyzed into glucose under acidic conditions and reacted with anthrone reagent to form a colored product, whose absorbance was measured at 620 nm. Trehalose content was determined using an enzymatic colorimetric method. Trehalose was specifically hydrolyzed into glucose by trehalase, and the glucose content was then detected using the GOPOD colorimetric system. Trehalose content was calculated after subtracting the background level of free glucose. The content of each carbohydrate was calculated based on the corresponding standard curve and expressed as mg/g FW.

### 4.5. RNA Extraction, Library Construction, and Transcriptome Sequencing

Based on the preliminary anatomical observations, six representative stages, namely ZH1, ZH2, BY3, BY4, ZH5, and ZH6, were selected for transcriptome sequencing, with three replicates per stage, resulting in a total of 18 samples. Total RNA was extracted using a TIANGEN polysaccharide- and polyphenol-rich plant total RNA extraction kit (DP862-T5C; Tiangen Biotech Co., Ltd., Beijing, China). RNA quality was assessed using a NanoDrop 2000 spectrophotometer (Thermo Fisher Scientific, Waltham, MA, USA), agarose gel electrophoresis, and an Agilent 5300 Fragment Analyzer system (Agilent Technologies, Santa Clara, CA, USA), and samples meeting the quality requirements (total amount ≥ 1 μg, concentration ≥ 30 ng/μL, RQN > 6.5, and OD260/280 = 1.8–2.2) were sent to Shanghai Majorbio Bio-pharm Technology Co., Ltd. (Shanghai, China)for library construction and sequencing. mRNA was enriched using Oligo(dT) magnetic beads, followed by cDNA library construction, and paired-end sequencing was performed on the DNBSEQ-T7 platform (MGI Tech Co., Ltd., Shenzhen, China).

### 4.6. Quality Control, Alignment, and Expression Quantification of Sequencing Data

Raw sequencing data were subjected to quality control using FastQC, and adapters and low-quality reads were removed using Trimmomatic. The clean reads were then aligned to the areca palm reference genome (*Areca catechu*, CNA0003279) [69] using HISAT2. Gene expression levels were quantified using RSEM, and a TPM (transcripts per million)-normalized expression matrix was generated.

### 4.7. Identification of Differentially Expressed Genes and Functional Enrichment Analysis

Differential expression analysis was performed on the raw gene count matrix using DESeq2 to identify differentially expressed genes between the degeneration window and the adjacent normally developing stages. The screening thresholds were set as an adjusted *p* value (padj) < 0.05 and |log_2_FC| ≥ 2, and multiple testing correction was performed using the Benjamini–Hochberg method [70]. Intersection analysis was conducted on the differentially expressed genes (DEGs) to obtain common DEGs consistently present in the degeneration window. Gene Ontology (GO) enrichment analysis was performed using GOATOOLS [71], and KEGG pathway enrichment analysis was conducted using the clusterProfiler package (v4.8.2) in R software (v4.3.1), with the Kyoto Encyclopedia of Genes and Genomes (KEGG) database used as the reference. An adjusted *p* value < 0.05 was considered the criterion for significant enrichment [72].

### 4.8. Weighted Gene Co-Expression Network Analysis

Weighted gene co-expression network analysis (WGCNA) was performed to investigate the co-expression patterns of genes associated with the degeneration window. The TPM expression matrix of 18 samples from six developmental stages was used as the input, and genes with an average expression level ≥ 1 and a coefficient of variation ≥ 0.1 were retained for network construction. Network construction was performed using the WGCNA package in R with the following parameters: signed network, soft-thresholding power β = 20, minModuleSize = 30, minKMEtoStay = 0.3, and mergeCutHeight = 0.25. Key modules significantly associated with traits were identified by Spearman correlation analysis (*p* < 0.05), and the top 30 genes with the highest intramodular connectivity within each module were selected as hub genes. For network visualization, only edges with weights > 0.02 were retained [73].

### 4.9. Quantitative Real-Time PCR

Total RNA was extracted using the same method as described for RNA-seq, and cDNA was synthesized using an Evo M-MLV reverse transcription kit (catalog no. AG11711; Accurate Biotechnology (Hunan) Co., Ltd., Hunan, China). qRT-PCR was performed on a Bio-Rad CFX96 system (Bio-Rad Laboratories, Hercules, CA, USA) using 2× SYBR Green Pro Taq HS Premix II (catalog no. AG11701; Accurate Biotechnology (Hunan) Co., Ltd., Changsha, China). The primer sequences are listed in Table S3. β-actin was used as the internal reference gene, and relative gene expression levels were calculated using the 2^−ΔΔCt method [74].

### 4.10. Data Processing and Analysis

Experimental data analysis and graphing were performed using GraphPad Prism 10.1.2. Results are presented as the mean ± standard deviation (mean ± SD). Differences among treatments were tested by one-way analysis of variance (one-way ANOVA), and multiple comparisons were performed using Tukey’s test. Differences were considered statistically significant at *p* < 0.05.

## 5. Conclusions

This study demonstrated that inflorescence degeneration in areca palm during the full-bloom stage does not occur randomly but is mainly concentrated at the BY3 and BY4 stages, and is characterized by typical phenotypic features, including reduced inflorescence size, tissue browning, decreased fresh weight, and developmental arrest. Compared with normally developing inflorescences, the degeneration window showed significant decreases in sucrose and starch contents, significant increases in ABA, JA, and MeJA levels, and a significant decrease in cZR content. Meanwhile, genes involved in starch and sucrose metabolism and plant hormone signal transduction underwent directional expression remodeling. Transcriptome analysis showed that the degeneration window was clearly separated from adjacent normally developing stages in the overall expression pattern, and a total of 2399 shared differentially expressed genes were identified. These genes were mainly enriched in starch and sucrose metabolism, plant hormone signal transduction, and transcriptional regulation-related processes. WGCNA identified modules significantly associated with the degeneration window and key physiological traits, and identified six hub genes, namely *AcAHP2*, *AcTIFY4B*, *AcTPS9-2*, *AcHXK2*, *AcWRKY3*, and *AcMPK1*. The qRT-PCR results further validated the reliability of the transcriptome data. These findings indicate that areca palm inflorescence degeneration during the full-bloom stage mainly occurs within a specific developmental window and is associated with carbon metabolic imbalance, hormone network remodeling, and key regulatory nodes at the co-expression network level. This study deepens our understanding of the mechanisms underlying areca palm inflorescence degeneration from the perspectives of developmental windows, physiological imbalance, and molecular networks, and provides a theoretical basis for early identification of degeneration, molecular regulatory studies, and improvement of degeneration-resistant germplasm.

## Data Availability

The data that support the findings of this study have been deposited into CNGB Sequence Archive (CNSA) [75] of China National GeneBank Database (CNGBdb) [76] with accession number CNP0009560.

## Supplementary Materials

The following supporting information can be downloaded at: https://www.mdpi.com/article/10.3390/plants15131962/s1, Figure S1: Schematic illustration of the leaf-position sequence and developmental states of axillary inflorescences in areca palm; Figure S2: Anatomical observation of areca inflorescence primordia at successive leaf positions during different phenological stages; Figure S3: Correlation and clustering analysis of RNA-seq samples from areca palm inflorescences at different developmental stages. Table S1: Significant differences in plant hormone contents; Table S2: Summary of RNA-seq quality and mapping statistics for all samples; Table S3: Real-time qPCR gene-specific primer sequences.

## Abbreviations

The following abbreviations are used in this manuscript:

ABA	abscisic acid
CK	cytokinin
DEGs	differentially expressed genes
GO	Gene Ontology
IAA	indole-3-acetic acid
JA	jasmonic acid
KEGG	Kyoto Encyclopedia of Genes and Genomes
MeJA	methyl jasmonate
MRM	multiple reaction monitoring
PCA	principal component analysis
RNA-seq	RNA sequencing
ROS	reactive oxygen species
TPM	transcripts per million
Tre6P	trehalose 6-phosphate
WGCNA	weighted gene co-expression network analysis
ZH	normal inflorescence
BY	degenerated inflorescence
