# Supplementary figures and images for: Physiological and Transcriptomic Dissection of Inflorescence Degeneration in Areca catechu L.: Aberrant Carbohydrate Redistribution and Disrupted Hormonal Homeostasis
Source: Plants (Basel). 2026 Jun 25;15(13):1962. doi: 10.3390/plants15131962 (PMC13364432; doi:10.3390/plants15131962)

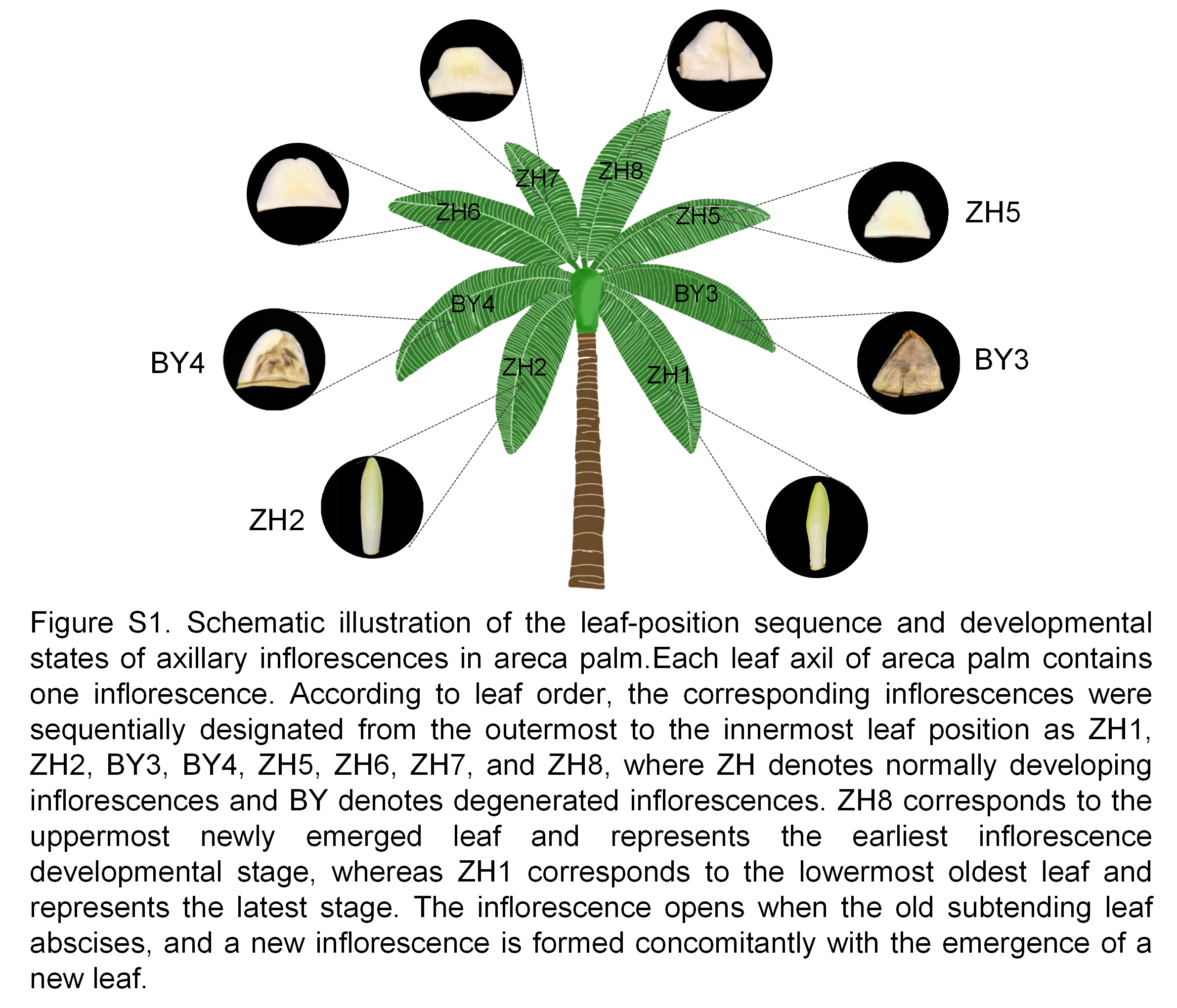

Supplement: Supplementary file 1 [file plants-15-01962-s001.zip › Figure S1_01.png]

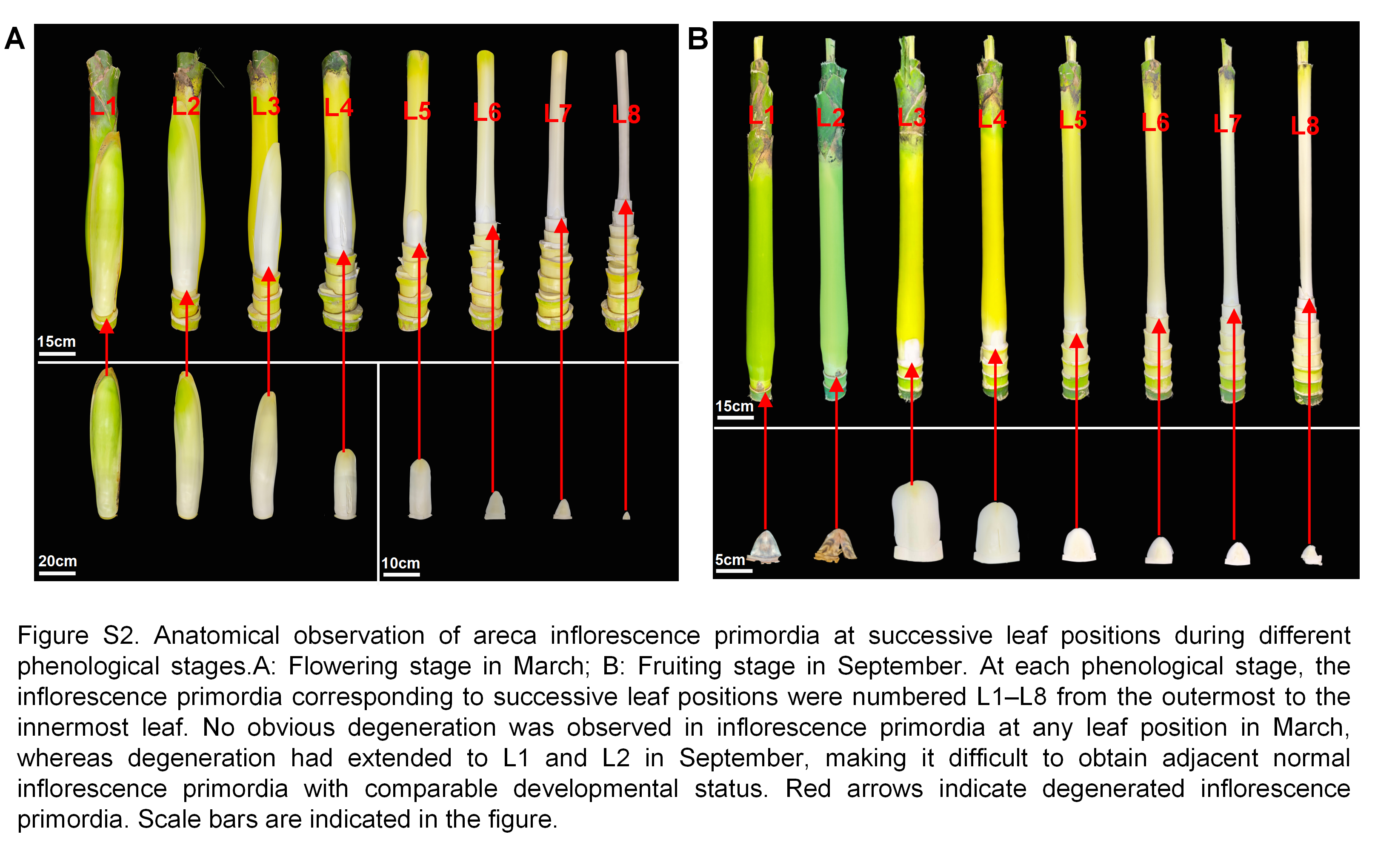

Supplement: Supplementary file 1 [file plants-15-01962-s001.zip › Figure S2_01.png]

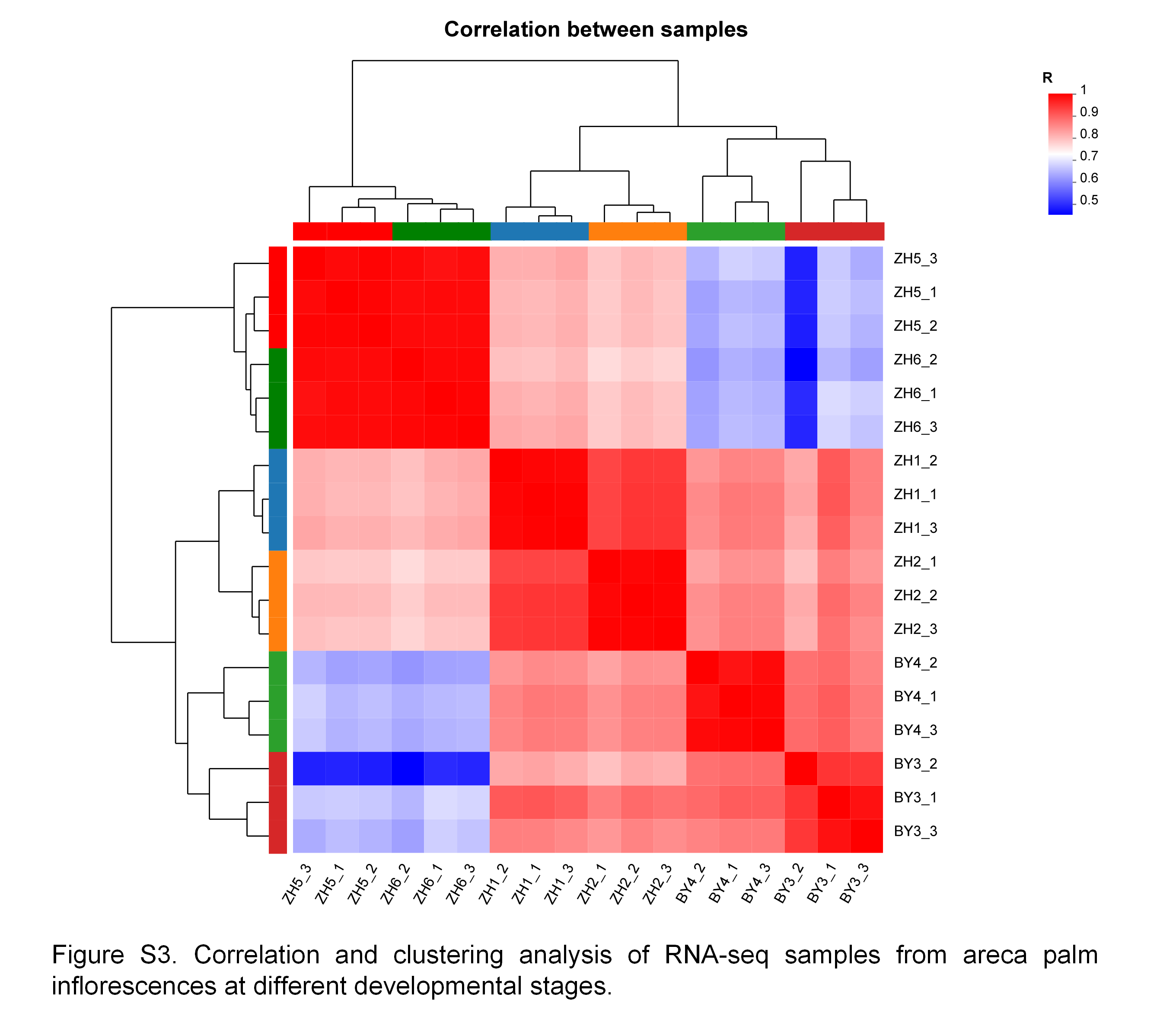

Supplement: Supplementary file 1 [file plants-15-01962-s001.zip › Figure S3_01.png]
